# Sialic acids as attachment factors in mosquitoes mediating Japanese encephalitis virus infection

**DOI:** 10.1128/jvi.01959-23

**Published:** 2024-04-18

**Authors:** Yi He, Chang Miao, Shiping Yang, Changhao Xu, Yuwei Liu, Xi Zhu, Yiping Wen, Rui Wu, Qin Zhao, Xiaobo Huang, Qigui Yan, Yifei Lang, Shan Zhao, Yiping Wang, Xinfeng Han, Sanjie Cao, Yajie Hu, Senyan Du

**Affiliations:** 1 Research Center for Swine Diseases, College of Veterinary Medicine, Sichuan Agricultural University, Chengdu, China; 2 Sichuan Science-Observation Experimental Station for Veterinary Drugs and Veterinary Diagnostic Technology, Ministry of Agriculture, Chengdu, China; 3 Engineering Research Center of Southwest Animal Disease Prevention and Control Technology, Ministry of Education of the People’s Republic of China, Chengdu, China; 4 Sichuan Center for Disease Control and Prevention, Chengdu, China; University Medical Center Freiburg, Freiburg, Germany

**Keywords:** Japanese encephalitis virus, sialic acids, infection in mosquitoes, attachment

## Abstract

The role of *Culex* mosquitoes in the transmission of Japanese encephalitis virus (JEV) is crucial, yet the mechanisms of JEV infection in these vectors remain unclear. Previous research has indicated that various host factors participate in JEV infection. Herein, we present evidence that mosquito sialic acids enhance JEV infection both *in vivo* and *in vitro*. By treating mosquitoes and C6/36 cells with neuraminidase or lectin, the function of sialic acids is effectively blocked, resulting in significant inhibition of JEV infection. Furthermore, knockdown of the sialic acid biosynthesis genes in *Culex* mosquitoes also leads to a reduction in JEV infection. Moreover, our research revealed that sialic acids play a role in the attachment of JEV to mosquito cells, but not in its internalization. To further explore the mechanisms underlying the promotion of JEV attachment by sialic acids, we conducted immunoprecipitation experiments to confirm the direct binding of sialic acids to the last α-helix in JEV envelope protein domain III. Overall, our study contributes to a molecular comprehension of the interaction between mosquitoes and JEV and offers potential strategies for preventing the dissemination of flavivirus in natural environments.

**IMPORTANCE:**

In this study, we aimed to investigate the impact of glycoconjugate sialic acids on mosquito infection with Japanese encephalitis virus (JEV). Our findings demonstrate that sialic acids play a crucial role in enhancing JEV infection by facilitating the attachment of the virus to the cell membrane. Furthermore, our investigation revealed that sialic acids directly bind to the final α-helix in the JEV envelope protein domain III, thereby accelerating virus adsorption. Collectively, our results highlight the significance of mosquito sialic acids in JEV infection within vectors, contributing to a better understanding of the interaction between mosquitoes and JEV.

## INTRODUCTION

Japanese encephalitis virus (JEV) is a zoonotic virus of the *Flavivirus* genus that is transmitted to humans and animals by *Culex tritaeniorhynchus* and *Culex quinquefasciatus. Aedes albopictus* is also a potential mosquito vector (1–3). JEV is the main cause of viral encephalitis in tropical and subtropical regions of Asia and the Pacific, posing a significant risk to more than 1,543.1 million individuals. The annual incidence of the virus in humans is in the range of 56,847 (4–7). Pigs infected with JEV exhibit age-related patterns, with piglets experiencing nonsuppurative encephalitis and adult pigs manifesting reproductive diseases such as abortions and transient infertility, adversely impacting food security (8, 9). The recent emergence of JEV in Australia has increased stillbirths, mummified fetuses, and neurological symptoms in piglets, as well as caused human cases. This poses a substantial public health concern and an agricultural economic threat (10–13). While vaccines for JEV exist, there are currently no curative drugs available. JEV follows a zoonotic transmission cycle between mosquito vectors and vertebrate hosts, with domestic pigs serving as amplification hosts for the virus. The role of mosquitoes in the transmission and infection of JEV is essential, but the mechanism by which JEV infects mosquito vectors has not been fully elucidated. A comprehensive understanding of the interaction between JEV and mosquito vectors is imperative for effective control of JEV transmission.

The virus enters cells through receptor-mediated endocytosis, and domain III of the E protein plays a key role in interacting with cellular receptors (14, 15). To date, numerous mammalian cell factors, including heparin sulfate, CD209/DC-SIGN, CLEC5A, HSP70, GRP78, integrins, and phosphatidylserine receptors such as TIM/TAM, have been proposed as potential JEV receptors (16–27). However, reports on the vector factors associated with JEV infection are limited. Previous research has indicated that GCTL-7 in *Aedes aegypti* can facilitate viral entry by binding to the JEV E protein at N154, although the *Aedes* genus is not the natural vector for JEV circulation (28). Additionally, the defensin of *Culex pipiens pallens* binds directly to domain III and promotes JEV entry by interacting with lipoprotein receptor-related protein 2 (29). However, other factors involved in *Culex* mosquito infection with JEV remain unclear.

Viruses employ various mechanisms to attach to or enter host cells, utilizing specific components on the cell membrane, such as glycans linked to proteins or lipids. Sialic acids, a group of sugars with a nine-carbon backbone, have been identified as essential receptors for many viral pathogens in vertebrate hosts, such as influenza A virus, parainfluenza virus, adenovirus, and coronavirus (30–35). However, recent studies have demonstrated that the sialylation pathways in both vertebrates and invertebrates, including *Drosophila melanogaster*, silkworm and mosquitoes, are similar (36–39). These data support the hypothesis that sialic acids might play vital roles in insect hosts of viruses. Previous research demonstrated that *Plasmodium gallinaceum* ookinetes recognized the midgut epithelium using sialic acids (40). Recent studies have shown that dengue virus (DENV) interacts with sialic acids in various *Aedes aegypti* tissues (41). Other research suggests that sialic acids in the *Culex pipiens* midgut may be involved in *Trypanosoma congolense* infection (42). Therefore, it is crucial to understand the role of sialic acids in the process of JEV infection.

This study revealed that sialic acids on the surface of mosquito cells facilitate JEV infection by serving as attachment factors for viral adsorption onto mosquito cells. Furthermore, our investigation demonstrated that sialic acids bind directly to the E protein domain III located at amino acids 402–426. These findings collectively suggest that sialic acids in mosquito vectors play a crucial role as attachment factors in JEV infection and might contribute to the transmission of the virus.

## RESULTS

### Blocking sialic acid on the mosquito vector cell surface with neuraminidase or lectin reduced JEV infection *in vitro* and *in vivo*

**Fig 1:**
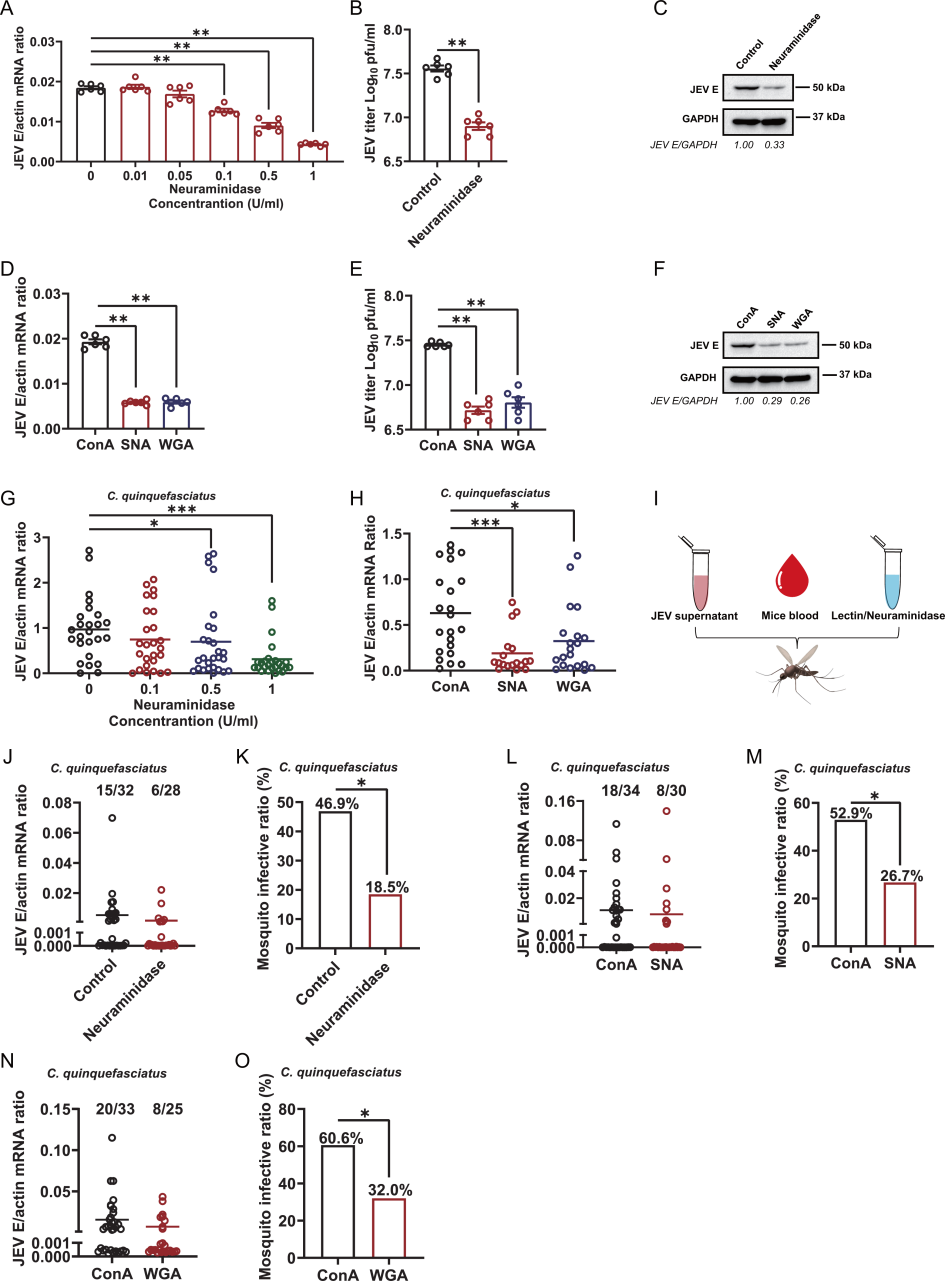
JEV infection was inhibited by blocking sialic acid on C6/36 cells and mosquito. (**A–F**) C6/36 cells were pretreated with increasing concentrations of neuraminidase from *C. perfringens* or 1 µM/mL lectins (ConA, SNA, and WGA), followed by JEV infection at a multiplicity of infection (MOI) of 0.5. (**A and D**) The infected cells were collected at 24 h post-infection for detection of viral genomes by RT-qPCR. (**B, C, E, and F**) C6/36 cells were pretreated with 1 U/mL neuraminidase or 1 µM/mL lectins, and the cell culture supernatant and infected cells were collected at 36 h post-infection. (**B and E**) Viral titers in the supernatant were determined by plaque assay. (**C and F**) JEV E protein abundance in infected cells was assessed by western blot assays using JEV E polyclonal antibody and GAPDH monoclonal antibody. (**G and H**) Increasing concentrations of neuraminidase or 2 µM/mL lectins were microinjected into *C. quinquefasciatus* thoraxes 1 day before 10 50% mosquito infectious dose (MID50) JEV infection. The JEV burden in entire mosquitoes was determined by RT-qPCR at 3 days post-infection. (**I**) Schematic of the study design. First, 1 U/mL neuraminidase, 2 µM/mL SNA, or 10 µM/mL WGA mixed with fresh mice blood and supernatant from JEV-infected C6/36 cells was used to feed *C. quinquefasciatus* via an *in vitro* blood feeding system. The JEV infection of entire mosquitoes was determined by RT-qPCR 8 days after a blood meal. (**J, L, and N**) The number of infected mosquitoes relative to the total number of mosquitoes is shown at the top of each column. Each dot represents a mosquito. The horizontal line represents the mean value of the group. The limit of detection for the viral genome/actin mRNA ratio was 0.001. Gene expression was normalized to the *C. quinquefasciatus* actin gene. The data are presented as the mean ± SEM. (**A, B, D, E, G, and H**) The nonparametric Mann-Whitney test was used for statistical analyses. (**K, M, and O**) The data at the top of each column represent the percentage of infected mosquitoes. Differences in the infectivity ratio were compared using Fisher’s exact test. *P < 0.05 and **P < 0.01. The experiment was repeated three times with similar results.

In mammals, sialic acids play a particularly prominent role in cell and molecular interactions with glycoproteins or glycolipids (43). Recent studies have investigated the biosynthesis of sialic acids in *Drosophila* and mosquitoes (41, 44, 45). Unlike vertebrates, in which sialic acids exist in distinct isoforms based on their linkage to the terminal glycoconjugates, insects such as *Drosophila* and mosquitoes exhibit only α2, 6-linked sialic acid structures on their surface (38). Sialic acids serve as attachment receptors for many viruses, suggesting that they play a crucial role in JEV infection in mosquito vectors. To investigate the physiological role of sialic acid in JEV infection, we first treated C6/36 cells, a mature mosquito cell line used for *in vitro* assays with titrated neuraminidases from *Clostridium perfringens*, which are sialidases that can remove cell surface sialic acid (46, 47). Then, we infected C6/36 cells with JEV. The infected cells were harvested at 24 h post-infection (hpi) to analyze the viral mRNA level through real-time quantitative polymerase chain reaction (RT-qPCR). JEV infection decreased with increasing concentrations of neuraminidase in a dose-dependent manner (Fig. 1A). We subsequently analyzed the viral burden in the supernatant by plaque assay and in the cell lysates by western blot at 36 hpi. The amount of virus particles in the supernatant decreased 4.53-fold after sialidase treatment (Fig. 1B). The protein levels of E in cell lysates also decreased (Fig. 1C). To further confirm the effect of sialic acid on JEV infection, C6/36 cells were pretreated with either of two lectins, SNA or WGA, which can specifically bind sialic acid (46, 48, 49), before JEV infection. ConA was used as a negative control. The two lectins significantly decreased the viral load, as measured by RT-qPCR, plaque assays, and western blotting, and the titer of the supernatant was reduced 5.4-fold and 4.42-fold, compared with that in the ConA treatment group (Fig. 1D through F).

To confirm that sialic acids mediate JEV infection *in vivo*, neuraminidases or lectins were microinjected into female *Culex quinquefasciatus* 1 day before JEV infection and virus levels were quantified by RT-qPCR at 3 days post-infection. One day after the injection of neuraminidase, we dissected the midguts of the mosquitoes and performed immunofluorescence staining. Notably, the level of sialic acids was significantly reduced (Fig. S5A). Compared with those in the control group, the levels of JEV E mRNA in the treatment groups were lower (Fig. 1G and H). Sialic acids are expressed on almost all extracellular glycans, including those in the midgut (41). JEV can overcome midgut barriers and then invade the whole body. We therefore investigated the role of sialic acids in the JEV acquisition process. *C. perfringens* neuraminidases or two lectins were fed via an *in vitro* blood feeding system containing blood and JEV (Fig. 1I). After 8 days of rearing, the fed mosquitoes were sacrificed and JEV infectivity was assessed by RT-qPCR. Compared with those in the control group, the infection ratios were decreased to 18.5%, 26.7%, and 32.0%, in *Culex quinquefasciatus* treated with neuraminidases or two lectins (Fig. 1J through O), which was consistent with the findings obtained after microinjection. *Aedes albopictus* is also considered a potential vector for JEV transmission. We next performed the same experiments to assess whether sialic acids in *Aedes albopictus* regulates JEV infection. Similar to previous results, JEV infection was significantly affected by the reduction of sialic acids on the cell surface by neuraminidases or lectins (Fig. S1A through F). These results indicated that sialic acids play an important role in JEV infection in mosquito species.

### Sialic acid synthetase knockdown inhibits JEV infection in *Culex quinquefasciatus*

**Fig 2:**
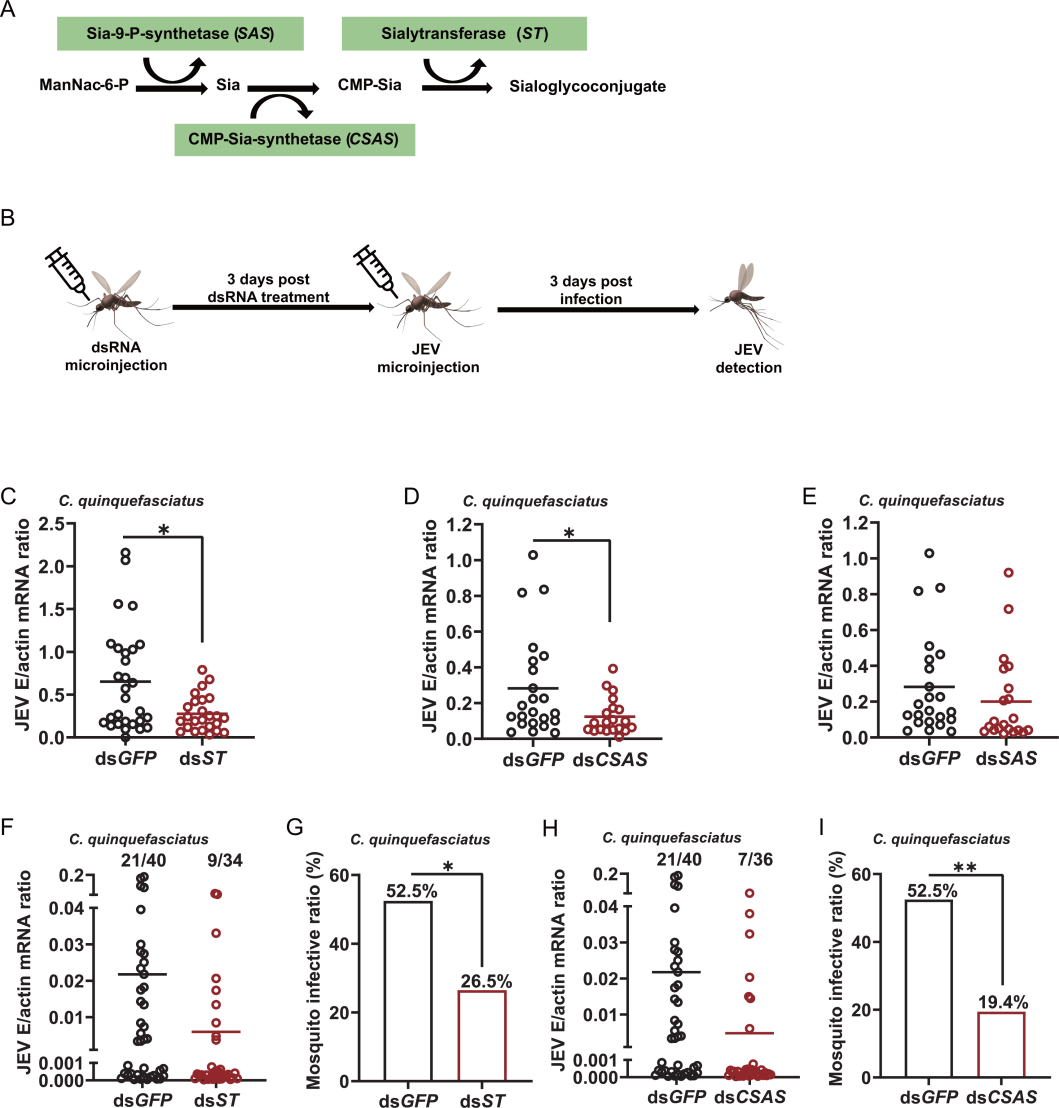
The sialic acid biosynthetic pathway was correlated with JEV infection. (**A**) Schematic of the sialic acid biosynthetic pathway in insects. (**B**) Schematic representation of the study design. The *CSAS*, *ST*, or *SAS* gene was silenced by thoracic microinjection of dsRNA in *C. quinquefasciatus*. The mosquitoes were inoculated with *GFP* dsRNA as mock controls. After 3 days, the post-dsRNA treatment mosquitoes were microinjected with 10 MID50 JEV. The entire mosquitoes were killed for JEV detection at 3 days post-infection. (**C–E**) The JEV burden in microinjected mosquitoes was determined at 3 days post-infection. Each dot represents the mRNA level in one mosquito. The horizontal line represents the median. The data are presented as the mean ± SEM. The nonparametric Mann-Whitney test was used for statistical analysis. (**F–I**) After 3 days of dsRNA treatment, the mosquitoes were infected with JEV by membrane feeding. The infection rate of mosquitoes was determined after 8 days. (**F and H**) The number of infected mosquitoes relative to the total number of mosquitoes is shown at the top of each column. Each dot represents a mosquito. The horizontal line represents the mean value of the group. (**G and I**) The data at the top of each column represent the percentage of infected mosquitoes. Differences in the infectivity ratio were compared using Fisher’s exact test. *P < 0.05. The data from two independent experiments were combined.

Sialylation in insects has been confirmed and relies on three pivotal enzymes. Neu5Ac synthetase (*SAS*) can use ManMAc-6-P as a substrate to produce sia-9-P, which initiates the sialic acid biosynthetic pathway in insects. CMP-sialic acid synthetase (*CSAS*) is an evolutionarily conserved enzyme mediating a key step in the biosynthetic pathway of sialylation and products CMP-sialic acid. CMP-sialic acid serves as a substrate for sialyltransferase (*ST*) to modify the termini of glycan binding on glycoproteins or glycolipids as the final step in the biosynthetic pathway (38, 50) (Fig. 2A). Therefore, we investigated the relationship between sialic acid synthetases and JEV infection in *Culex quinquefasciatus* over the course of 6 days of infection. Female mosquitoes were infected by administration of 100 MID50 by microinjection. As shown in Fig. S2A through C, with JEV infection, except the *ST*, the *SAS* and *CSAS* mRNA abundances were increased after 6 days of infection, indicating that JEV regulated the biosynthesis of sialic acids. To further examine the function of sialic acid in JEV infection, double-stranded RNA-mediated silencing in mosquitoes was employed. DsRNA for *CSAS*, *ST*, and *SAS* were synthesized and individually microinjected into female *Culex quinquefasciatus*. JEV was sequentially inoculated 3 days later, and the effect on viral burden was assessed 3 days after infection (Fig. 2B). Compared with the *GFP* dsRNA-inoculated control, knockdown of *CSAS* and *ST* genes significantly decreased the level of sialic acids and the JEV burden in mosquitoes (*P* < 0.05) (Fig. S5B; Fig. 2C and D). In contrast, suppression of the *SAS* gene did not influence the JEV load (Fig. 2E). Moreover, the infective ratio after blood feeding was also decreased to 26.5% and 19.4%, respectively, in *Culex quinquefasciatus* by silencing the *CSAS* and *ST* genes (Fig. 2F through I). At the same time, we determined the expression of *CSAS*, *ST*, and *SAS* after gene silencing. The expression of the three genes was reduced 4- to 5-fold compared with that in the control (Supplementary Fig. S2D through F), indicating that the impairment of JEV infection was correlated with dsRNA inoculation. These results suggested that the sialic acid biosynthetic pathway was correlated with JEV infection.

### Sialic acid on mosquito cell surface facilitates JEV attachment, not internalization

**Fig 3:**
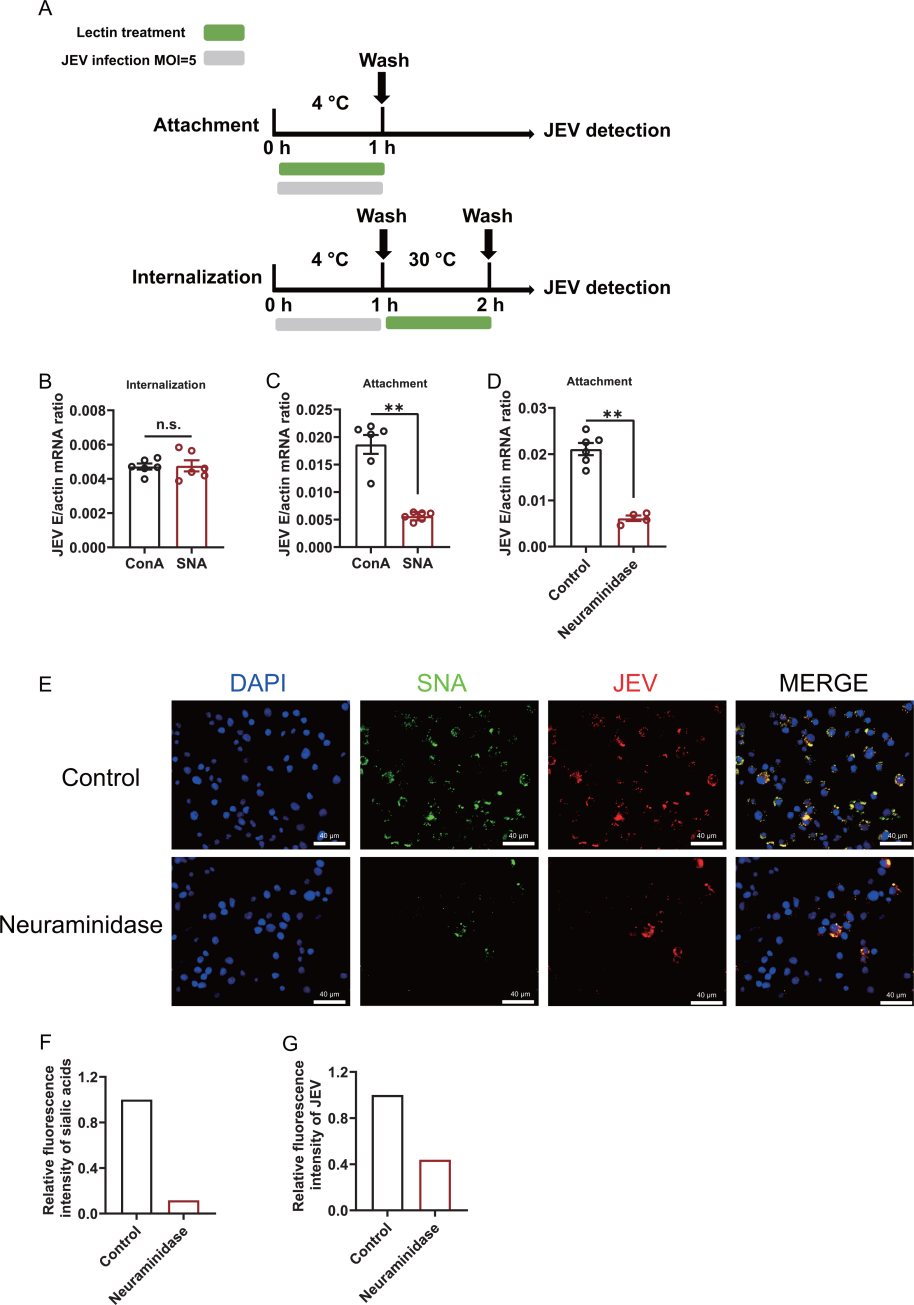
Sialic acids promote JEV attachment. (**A**) Schematic representation of the study design. For the attachment assay, C6/36 cells were incubated with JEV at a MOI of 5 and 1 µM/mL lectins (ConA or SNA) for 1 h at 4°C. The unbound virions were removed by washing with PBS. (**C**) The cell extracts were subjected to RT-qPCR. For the internalization assay, after JEV virion adsorption at 4°C for 1 h, the cells were washed with PBS and subsequently incubated to 30°C for 1 h with 1 µM/mL lectins (ConA or SNA) to allow virion internalization. After washing with PBS, the cells were treated with sodium citrate buffer to remove noninternalized virions. (**B**) Viral internalization into cells was assessed by RT-qPCR. (**D and E**) After treatment with 1 U/mL neuraminidase, C6/36 cells were infected with JEV at a MOI of 5 at 4°C for 1 h. Unbound viruses were removed by washing with PBS. (**D**) Viral attachment was assessed by RT-qPCR. (**E**) Immunofluorescence analysis. Cells were stained with an anti-JEV E polyclonal antibody (red) and biotin-labeled SNA (green), and the nuclei were stained with DAPI. Scale bars, 40 µm. (**F and G**) The relative fluorescence intensities of sialic acids and JEV were calculated by ImageJ. (**B–D**) The data are presented as the mean ± SEM. The nonparametric Mann-Whitney test was used for statistical analysis. **P < 0.01; n.s., not significant.

**Fig 4:**
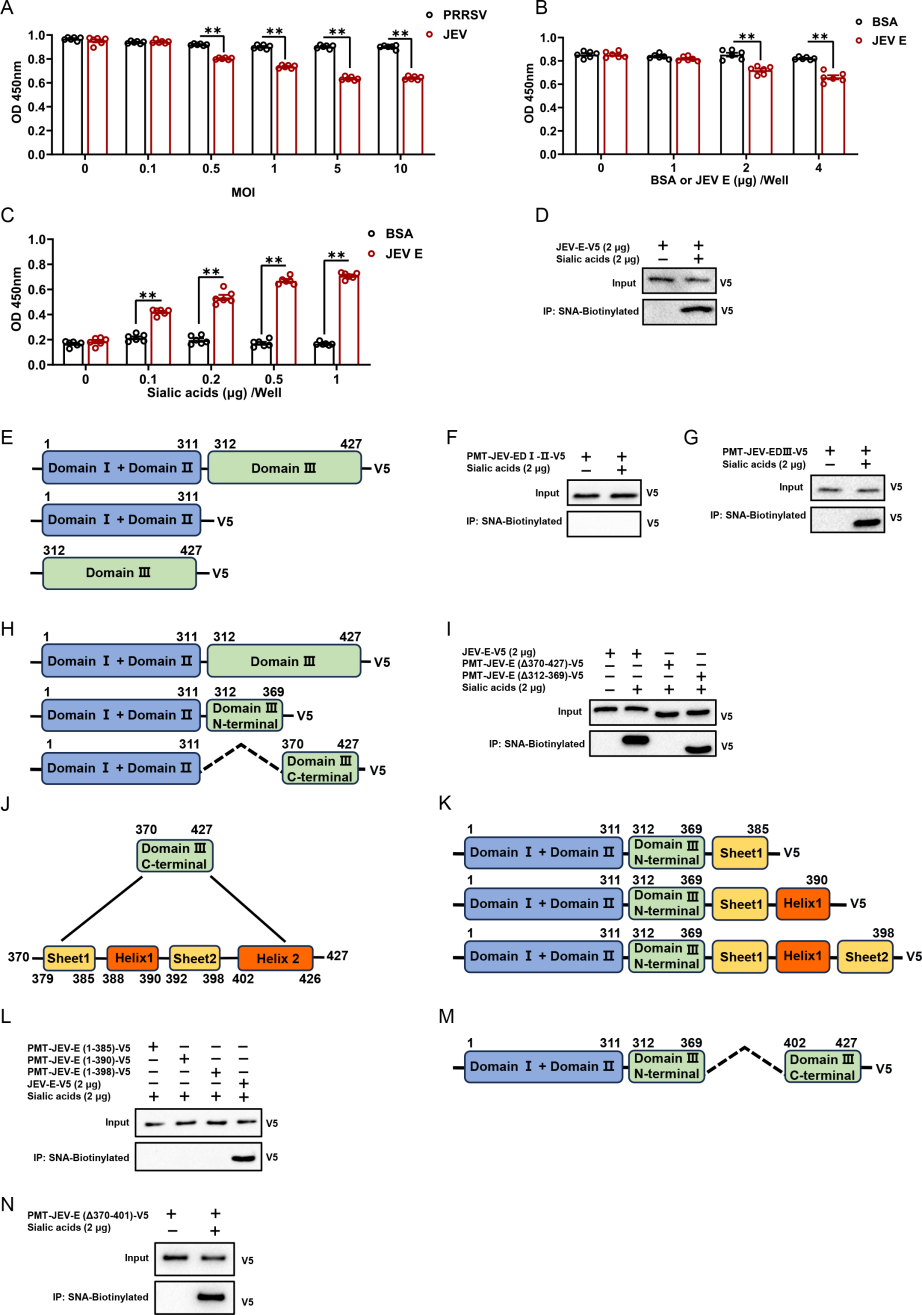
Sialic acids interact with the JEV E protein at the last α-helix in domain III. (**A and B**) C6/36 cells were seeded into 96-well plates, and cells were incubated with virions or protein. Sialic acids on the C6/36 cell surface were bound to biotinylated SNA followed by HRP-conjugated streptavidin. The interaction of JEV virions or purified JEV E protein with sialic acids on the C6/36 cell surface was analyzed by cell-based ELISA. (**C**) Purified JEV E proteins were coated on an enzyme-labeled plate and incubated with different concentrations of sialic acid. The interaction between these proteins was measured by ELISA using biotinylated SNA followed by HRP-conjugated streptavidin. (**D, F, G, I, L, and N**) Purified JEV E protein or truncated E proteins from S2 cells that were transfected with recombinant plasmid (tagged with V5) were incubated with sialic acids. The protein complex was pulled down with biotinylated SNA. Bound proteins were eluted and analyzed by immunoblotting with a V5-tag antibody. (**E**) Schematic representation of the three domains of the JEV E protein. (**H**) Schematic representation of the truncations at the N-terminal and C-terminal of JEV E DIII; the dashed line means delete the N-termina of E DIII (Δ312–369 amino acids). (**J**) Schematic diagrams displaying the structure of the C-terminal of JEV E DIII (370–427 amino acids). (**K**) Schematic diagram showing the truncations of JEV E DIII (370–427 amino acids). (**M**) Schematic diagrams displaying the structure of JEV E (Δ370–401 amino acids); the dashed line means delete 370–401 amino acids. The data are presented as the mean ± SEM. The nonparametric Mann-Whitney test was used for statistical analysis. **P < 0.01.

Sialic acid is well known to act as an attachment or entry receptor for multiple viruses. To characterize the mechanism by which sialic acid assists JEV infection, we treated C6/36 cells with SNA 2 h before (pre), simultaneously with (co), or 2 h after (post) JEV incubation to identify the stage of JEV infection in which sialic acids act (Fig. S3A). As the results showed, post-treatment had no effect; however, pre-treatment and co-treatment led to a dramatic reduction in the amount of viral RNA (Fig. S3B), indicating that sialic acids might participate in the initial stages of virus infection (51). To investigate the effect of sialic acids on JEV attachment, C6/36 cells were incubated with JEV at 4°C for 1 h with or without SNA. Afterward, any unbound virions were removed by washing with phosphate-buffered saline (PBS). To assess the effect on internalization, C6/36 cells were incubated with JEV at 4°C for 1 h first to allow virion adsorption; then, the unbound virions were washed away with sodium citrate buffer (pH3.0) and subsequently shifted to 30°C for 1 h in the presence or absence of SNA (52, 53) (Fig. 3A). As shown in Fig. 3B, SNA did not interfere with JEV internalization into host cells. Nonetheless, viral attachment was significantly reduced by SNA (Fig. 3C). In agreement with lectin treatment, removal of cell surface sialic acid by neuraminidase also had the same inhibitory effect on JEV attachment at the mRNA level (Fig. 3D). We also performed immunofluorescence staining to further explore the effect of virus attachment after sialic acid removal by neuraminidase. C6/36 cells were treated with neuraminidase and then incubated with JEV at 4°C. Biotin-labeled SNA was not detected after neuraminidase treatment in C6/36 cells, indicating that sialic acids were removed and the JEV virions attached to the cell surface were stained with a polyclonal antibody generated in mice against JEV envelope protein (Fig. 4A and B). Without sialic acids, JEV binding to C6/36 cells was eliminated (Fig. 3E through G). This finding indicates that JEV attachment is dependent on sialic acid at least in part.

### Sialic acids promote JEV binding to mosquito cells by interacting with the E protein at the last α-helix in domain III

Next, we studied the exact mechanisms by which sialic acids promote JEV attachment. Based on previous results, we deduced that JEV might bind sialic acids. Therefore, the interaction between JEV and sialic acids was confirmed by enzyme-linked immunosorbent assay (ELISA). The wells of a plate were coated with C6/36 cells and incubated with JEV virions at different MOIs or with purified E protein expressed in *Drosophila* S2 cells (Fig. S4C); the virions of porcine reproductive and respiratory syndrome virus (PRRSV) and bovine serum albumin (BSA) were used separately as negative controls. Then, the plates were incubated with biotinylated SNA. As expected, as the concentration of virions or E protein increased, the amount of SNA binding to sialic acids on the cell surface decreased, which indicated that the JEV E protein and SNA competed for interaction with sialic acids (Fig. 4A and B). To analyze the direct interaction between the E protein and sialic acids, the wells of a plate were coated with the E protein, incubated with different concentrations of sialic acids, and assessed with biotinylated SNA. The results demonstrated that sialic acids could bind directly to the E protein in a dose-dependent manner (Fig. 4C). To verify this result, an IP experiment was performed. This result was consistent with our ELISA data (Fig. 4D). Therefore, all the results indicated that the E protein can efficiently bind sialic acids.

Each E protein ectodomain comprises three distinct domains: central β barrel domain I (DI), elongated finger-like domain II (DII), and immunoglobin like domain III (DIII) (14, 15). To provide a precise demonstration of the interaction between the E protein and sialic acids, the overexpression of DI-II and DIII in S2 cells was conducted separately (Fig. 4E), followed by an examination of the interaction with sialic acids. Sialic acids do not bind to DI-DII but exhibit strong interaction with DIII (Fig. 4F and G). To further examine the specific section of DIII interacting with sialic acids, two truncated E proteins were expressed: one with the C-terminal of DIII removed (Δ370–427 amino acids) and the other with the N-terminal of DIII deleted (Δ312–369 amino acids) (Fig. 4H). Our findings revealed that sialic acids interacted exclusively with the C-terminal region of DIII (370–427 amino acids) (Fig. 4I). Subsequently, we attempted to pinpoint the precise interaction region within amino acids 370–427. Analysis of the C-terminal structure, as depicted in Fig. 4J, revealed the presence of various sheets and helices. Based on this structural information, we constructed three V5-tagged truncated JEV E plasmids containing amino acids 1–385, 1–390, and 1–398 (Fig. 4K), which were then subjected to IP experiments to investigate their interactions with sialic acids. As shown in Fig. 4L, in contrast to the full-length E protein, all the truncated mutants exhibited deficiencies in interactions with sialic acids, indicating that the binding site encompasses amino acids 402–426. The subsequent generation of an additional truncated mutant (Δ370–401 amino acids) (Fig. 4M) enabled the further identification of the residues 402–426 as the region that interacted with sialic acids (Fig. 4N). These findings indicated that the last α-helix within DIII can bind directly to sialic acids, thereby facilitating JEV infection of mosquito vectors.

## DISCUSSION

Japanese encephalitis virus is a typical mosquito-borne zoonotic flavivirus that is accountable for inducing acute encephalitis and meningitis in humans (54), as well as fatal encephalitis, abortion, and stillbirth in pregnant sows (55, 56). Consequently, JEV poses a significant threat to public health and has adverse impacts on the development of animal husbandry. Currently, the primary approach for JEV control relies heavily on vaccination. However, due to the effects of global warming and urbanization, the geographical range of JEV is expanding, leading to a surge in epidemic occurrences in countries such as Pakistan and Australia (57, 58). *Culex* mosquitoes, in particular, serve as the primary vector for the transmission of JEV; however, the precise mechanism by which the virus infects these mosquitoes remains elusive. Therefore, it is imperative to elucidate the interaction between JEV and mosquito vectors to effectively manage mosquito-borne diseases. Our research has made a novel discovery, demonstrating the significant involvement of sialic acids in mosquitoes in JEV infection through their interaction with the E protein. This finding further uncovers the association between vectors and the virus.

Sialic acids, which are derivatives of neuraminic acid and consist of 9-carbon monosaccharides, are commonly found as terminal monosaccharides on various cell surfaces and secreted glycoconjugates (59). These sialic acids have been shown to facilitate the infection of mammals by different types of viruses, including influenza virus, coronavirus, adenovirus, and zika virus (ZIKV) (48, 60–63). The underlying mechanism by which sialic acids promote the infection process of these viruses has been gradually clarified. Interestingly, sialic acid is present not only in mammals but also in mosquitoes (41, 64). Therefore, we hypothesize that sialic acids in mosquitoes may play a role in JEV infection.

In our study, we observed that sialic acids play a crucial role in promoting JEV infection in both C6/36 cells and mosquitoes *in vitro* and *in vivo*. Notably, the application of neuraminidase or lectin to mosquitoes and C6/36 cells resulted in a significant inhibition of JEV infection. A similar effect was also observed in *Aedes albopictus*. Furthermore, the synthesis of sialic acids in insects involves three enzymes, namely, *SAS*, *CSAS*, and *ST* (38, 50). Following JEV infection in *Culex quinquefasciatus*, *SAS* and *CSAS* mRNA abundances were significantly upregulated. Importantly, the knockdown of *CSAS* and *ST* genes led to a significant reduction in JEV replicative capacity in mosquitoes. These results suggest that sialic acids are significant in the modulation of virus infection.

According to previous reports, sialic acids have been identified as potential virus attachment receptors that facilitate infection (62). Similarly, our study indicated that sialic acids can bind to the envelope protein of JEV to facilitate JEV attachment. Due to the intricate structure of the JEV E protein, we conducted additional experiments to determine which domain interacts with sialic acids. The findings consistently demonstrated that the last α-helix (residues 402–426) in domain III can interact with sialic acids. Collectively, these results provide compelling evidence that sialic acids are crucial auxiliary factors for JEV in mosquitoes. Nevertheless, given that the last α-helix in domain III comprises 25 amino acids, additional confirmation of the precise binding site is warranted. Further research will involve molecular docking studies to analyze the potential binding patterns.

**Fig 5:**
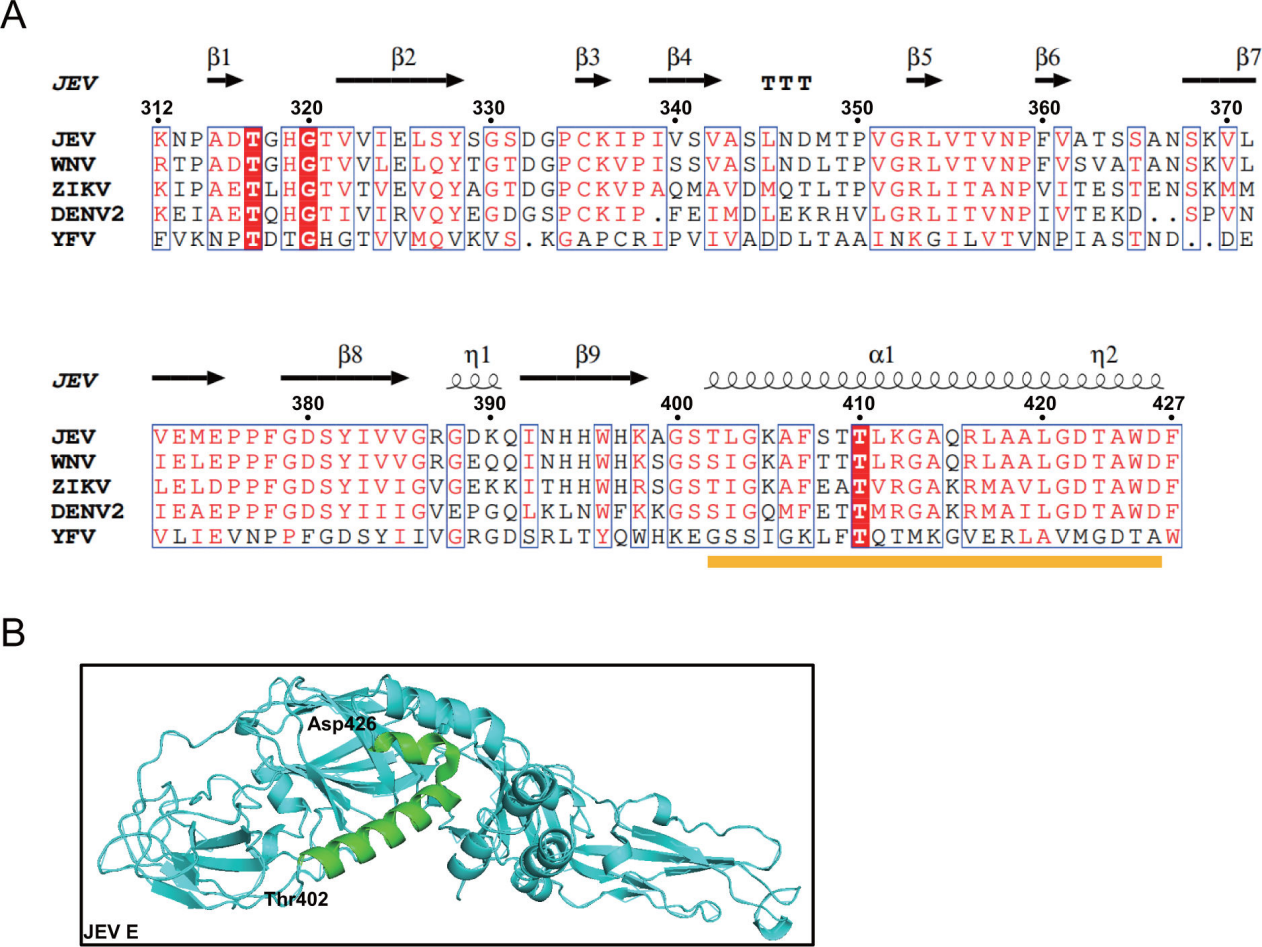
Alignment of the E DIII protein sequences of flaviviruses and the physical structure of the JEV E protein. (**A**) Alignment of the E DIII protein sequences of JEV, WNV, ZIKV, DENV, and YFV. The yellow line marks the last helix in DIII. (**B**) Model of the location of the last helix (402–426 amino acids) of the JEV E protein.

Mammals possess a variety of sialyltransferases responsible for synthesizing distinct sialic acid linkages in mammals, including α2, 3-linked sialic acid, α2, 6-linked sialic acid, and α2, 8-linked sialic acid (65). In contrast, insects possess only one sialyltransferase capable of synthesizing α2, 6-linked sialic acid (66). The lectin SNA specifically recognizes α2, 6-linked sialic acids, while WGA interacts with sialic acids of all linkages (63, 67). Our research demonstrated that both SNA and WGA significantly reduce JEV infection in C6/36 cells and mosquitoes, both *in vitro* and *in vivo*, by obstructing sialic acids. Despite the different linkages in arthropods and mammals, previous research has indicated that DENV interacts with sialic acids within the tissues of *Aedes aegypti*, and α2, 3-linked sialic acids play a role in the internalization of ZIKV in Vero cells (41, 48). These findings imply that sialic acids may facilitate flavivirus infection in both mammals and mosquitoes through distinct mechanisms. These contrasting responses in mammals and mosquitoes require further research to better understand the functional divergence between different species of sialic acids. In light of the conservation of flavivirus envelope proteins, a comparative analysis was conducted on the envelope proteins of multiple flaviviruses. The physical structures of residues 402–426 in the E protein are highlighted in green. Notably, the last α-helix in the envelope protein is clearly conserved in JEV, DENV, ZIKV, West Nile virus (WNV), and Yellow fever virus (YFV) (Fig. 5A and B). This conservation implies that targeting sialic acids might be able to impede the transmission of various mosquito-borne viruses. The conserved nature of this structural element suggests a potential role for sialic acid in the pathogenesis of multiple flaviviruses, warranting additional research to elucidate this function.

The initial stage of JEV infection involves the attachment of the virus particles to sialic acids or other receptors on the cellular membrane, followed by internalization. However, the precise mechanism by which sialic acid-dependent attachment occurs remains unclear. It is worth noting that sialic acids are incapable of transmitting signals through the plasma membrane, suggesting the involvement of additional signaling molecules (68). Further research is needed to determine whether specific molecules are responsible for facilitating the process of JEV virion attachment to sialic acids across the plasma membrane.

In summary, our findings indicate that the interaction between mosquito sialic acids and JEV E protein can facilitate JEV infection. This research contributes novel perspectives to the prevention of JEV and enhances our comprehension of the mechanism underlying flavivirus infection in mosquitoes.

## MATERIALS AND METHODS

### Mosquitoes, cells, and virus

*C. quinquefasciatus* (the Chengdu strain) and *A. albopictus* (the Guangzhou strain) mosquitoes were reared on a sugar solution at 28°C and 80% humidity, according to standard rearing procedures (69–71). The BHK-21 cells and C6/36 cells were cultured in Dulbecco’s modified Eagle’s medium (Gibco) supplemented with 10% fetal bovine serum (ExCell Bio) and 1% antibiotic-antimycotic (Thermo). The *Drosophila melanogaster* S2 cell line was cultured in Schneider’s medium (Gibco) with 10% fetal bovine serum and 1% antibiotic-antimycotic. The BHK-21 and C6/36 cell lines were purchased from ATCC. The *Drosophila* S2 cell line was provided by Invitrogen. JEV (SA14 strain, MK585066.1) was maintained in our laboratory and propagated in C6/36 cells. Viral titer was assessed by a plaque assay titrated on BHK-21 cells. The virus for *in vivo* experiments was titrated by MID50 (69, 72). Experiments involving infectious virus were conducted in a Biosafety Level 2 laboratory.

### Western blotting analysis and antibodies

The JEV-E gene was amplified from the cDNA of infected cells and inserted into a pET-28a (+) expression vector (Millipore). The cloning primers are presented in Table S1. Recombinant JEV-E protein was expressed in the *Escherichia coli* BL21 DE3 strain in an insoluble form in inclusion bodies. The protein was then solubilized in 8 M urea and purified with a TALON Purification Kit (Clontech). Murine antiserum was produced via inoculation with recombinant E protein with three boosts, and the serum was divided and stored at −80°C until further use. The samples were separated by 12% SDS-PAGE gel, followed by electrophoretic transfer to polyvinylidene fluoride membranes and blocking and incubating membranes with primary antibodies. The following antibodies were used in these experiments: anti-V5-HRP (Invitrogen) antibodies and anti-GAPDH (Proteintech) antibodies.

### Expression and purification of JEV-E protein in the *Drosophila* expression system

The JEV E gene was cloned into a pMT/BiP/V5-His A vector (Invitrogen) for expression in *Drosophila* S2 cells. The cloning primers are shown in Table S1. The procedure used to generate a stable cell line is described in the manual of the *Drosophila* expression system (Invitrogen). For JEV E expressing, the stable S2 cells were cultured in Schneider’s medium in a 225-cm^2^ flask. After 3 days, the medium was replaced with Express Five serum-free medium (Gibco) for protein expression. The cells were cultured for 3 days and induced with 500 µM copper sulfate for another 4 days. The supernatant was centrifuged, filtered, and then concentrated for purification using a TALON Purification Kit (Clontech). Protein purity was verified by SDS-PAGE and immunostaining with an anti-V5 mouse antibody (69).

### Gene silencing and viral infection in mosquitoes

Detailed procedures for gene silencing in mosquitoes have been described before (73, 74). The primers for double-stranded RNA (dsRNA) synthesis are shown in Table S1. In brief, female mosquitoes were anesthetized in a cold tray, and 1 µg/300 nL of dsRNA was microinjected into their thoraxes. After a 3-day recovery period, the mosquitoes were microinjected with JEV 10 MID50/300 nL for functional studies. The gene silencing efficiency and JEV burden were assessed by RT-qPCR (75). The primers used for gene detection are shown in Table S1.

### Membrane *in vitro* blood feeding

Fresh mouse blood was placed in heparin-coated tubes and centrifuged at 1,000 × *g* for 10 minutes at 4°C to separate the plasma from the blood cells. The plasma was collected and heat inactivated at 55°C for 60 minutes. The separated blood cells were washed three times with PBS to remove the anticoagulant. Cells were then resuspended in heat-inactivated plasma. Either lectins or neuraminidase was mixed with JEV-infected supernatant from C6/36 cells and mice blood for mosquito oral feeding via a Hemotek system (Hemotek Limited). Engorged female mosquitoes were transferred into new containers and maintained under standard conditions for 8 days. The mosquitoes were subsequently euthanized for further investigations (76).

### Block and removal of cell surface sialic acid by lectin and neuraminidase

C6/36 cells in 24-well plates were treated with 1 µmol/mL ConA, SNA, and WGA lectins for 2 h at 28°C or increasing concentrations of neuraminidase for 3 h at 37°C (48, 67, 77) followed by washing with PBS. Lectin-treated cells were then infected with JEV at MOI of 0.5 for 2 h at 28°C. They were then washed to remove unbound virions and cultured with 2% FBS DMEM at 28°C for the indicated times. Cell lysates and supernatant virus were then collected for further analysis.

### Virus attachment and internalization assay

For the attachment assay, C6/36 cells were incubated with JEV at a MOI of 5 at 4°C for 1 h. Unbound JEV virions were removed by washing with PBS three times. JEV internalization assays were carried out by incubating JEV with C6/36 cells at 4°C for 1 h to allow JEV binding, after which the unbound JEV virions were removed by rapidly washing three times with PBS. Then, the cells were transferred to 30°C for 1 h to allow JEV internalization and treated with sodium citrate buffer (pH3.0) to remove noninternalized virions (78, 79). Total RNA was extracted, and the viral mRNA levels were determined by RT-qPCR.

### qPCR detection

The total RNA in cells and mosquitoes was extracted by the Total RNA Extraction Kit (AXYGEN), and cDNA was synthesized using a cDNA Reverse Transcription Kit (TRANSGEN), and qPCR was performed on the Bio-Rad CFX-96 Touch Real-Time Detection System. The primers and probes used are shown in Table S1. Gene expression was normalized to the mosquito actin gene.

### Co-immunoprecipitation

S2 cells were transfected with recombinant plasmids and induced with 500 µM copper sulfate for 2 days. The cells were harvested and incubated with IP lysis buffer supplemented with protease inhibitor cocktail. The cell lysates were incubated with 2 µg sialic acids (Solarbio) at 4°C overnight, and subsequently, the mixture was incubated with 10 µg/mL biotinylated SNA. Dynabeads MyOne Streptavidin T1 (Invitrogen) was added for 2 h incubation. After washing with IP lysis buffer, the samples were detected by western blotting.

### Immunofluorescence staining and microscopy

For JEV binding assays, C6/36 cells were seeded in 24-well plates containing coverslips. After JEV attachment, the cells were washed with PBS and fixed with 4% paraformaldehyde for 1 h. Cells were then blocked in 1% BSA for 1 h. JEV envelope antigens were immunostained with primary anti-JEV envelope polyclonal antibody at 1:200 at 4°C dilution and secondary Alexa Fluor 647-conjugated anti mouse IgG antibody (Abcam) at 1:200 dilution for 1 h at room temperature. For sialic acid staining, biotinylated SNA (Vector labs) was used to stain α2, 6-linked sialic acid at 1:200 dilution for 1 h at room temperature. FITC-conjugated streptavidin (Solarbio) was added at a 1:200 dilution for 1 h at room temperature. Cell nuclei were stained with DAPI (Beyotime). Images were examined using an Echo Revolve microscope.

### Purification of infectious JEV virions

Supernatants from JEV-infected C6/36 cells were collected 5 days after infection. Cell fragments were removed by centrifugation at 1,000 × *g* and 4°C for 10 minutes. Subsequently, the supernatant was carefully transferred into a clean centrifuge tube and centrifuged at 30,000 × *g* and 4°C for an additional 4 h to pellet the virions. The precipitated virions were solubilized in serum-free DMEM. The virions were aliquoted and stored at −80°C (80).

### Enzyme-linked immunosorbent assay

The purified E protein was used in an enzyme-linked immunosorbent assay. The E protein (1 µg) was coated overnight at 4°C. The plates were blocked using 5% wt/vol BSA solution for 1 h at room temperature. The different concentrations of sialic acids (Solarbio) were added and incubated for 2 h. After washing, biotinylated SNA was added and incubated for 2 h. After washing again, HRP-conjugated streptavidin (Proteintech) was added to each well, and the plates were incubated for 1 h. The commercial peroxidase substrate system was used for signaling detection (SeraCare), and the optical density (OD) at 450 nm was measured with an ELISA reader.

### Cell-based ELISA

C6/36 cells grown in a 96-well plate were incubated with JEV virions or purified JEV envelope protein. After 1 h at 4°C, the cells were washed with PBS and then fixed with 4% paraformaldehyde for 1 h. The cells were then blocked in 1% BSA for 1 h. Biotinylated SNA was used to stain α2, 6-linked sialic acid. After incubation, the cells were washed with PBST, and HRP-conjugated streptavidin was added. TMB reaction buffer was added for the colorimetric assays, and the signal was read at OD 450 nm (81).

### Sequence alignment by the program ESPript

The protein sequences of flaviviruses were retrieved from the NCBI database in FASTA format. Multiple sequence alignment and comparative analysis of the protein were carried out using ESPript, which can help optimize an alignment by displaying the secondary structure information of each aligned sequence in the same figure (82, 83). ESPript can be accessed via its website at https://espript.ibcp.fr/ESPript/ESPript/.

### Structure modeling

SWISS-MODEL was used to generate the structure of the JEV E protein and can be accessed via its website at SWISS-MODEL (expasy.org) (84). PyMOL (The PyMOL Molecular Graphics System, Version 2.0 Schrödinger, LLC. PyMOL | pymol.org) was used to analyze and render images of the structures (85).

### Statistical analysis

Animals were randomly allocated into different groups. Mosquitoes that died before counting and measurement were excluded from the analysis. The investigators were not blinded to the allocation during the experiments or to the outcome assessment. Descriptive statistics are provided in the figure legends. All analyses were performed using GraphPad Prism 8.0 statistical software.

## Data Availability

All data generated are included in the article.

## SUPPLEMENTAL MATERIAL

Supplemental material jvi.01959-23-s0001.docx Figures S1 to S5; Table S1.

ASM does not own the copyrights to Supplemental Material that may be linked to, or accessed through, an article. The authors have granted ASM a non-exclusive, world-wide license to publish the Supplemental Material files. Please contact the corresponding author directly for reuse.
