# Supplementary material for: Sialic acids as attachment factors in mosquitoes mediating Japanese encephalitis virus infection
Source: J Virol. 2024 Apr 18;98(5):e01959-23. doi: 10.1128/jvi.01959-23 (PMC11092328; doi:10.1128/jvi.01959-23)

**Supplementary information**

**
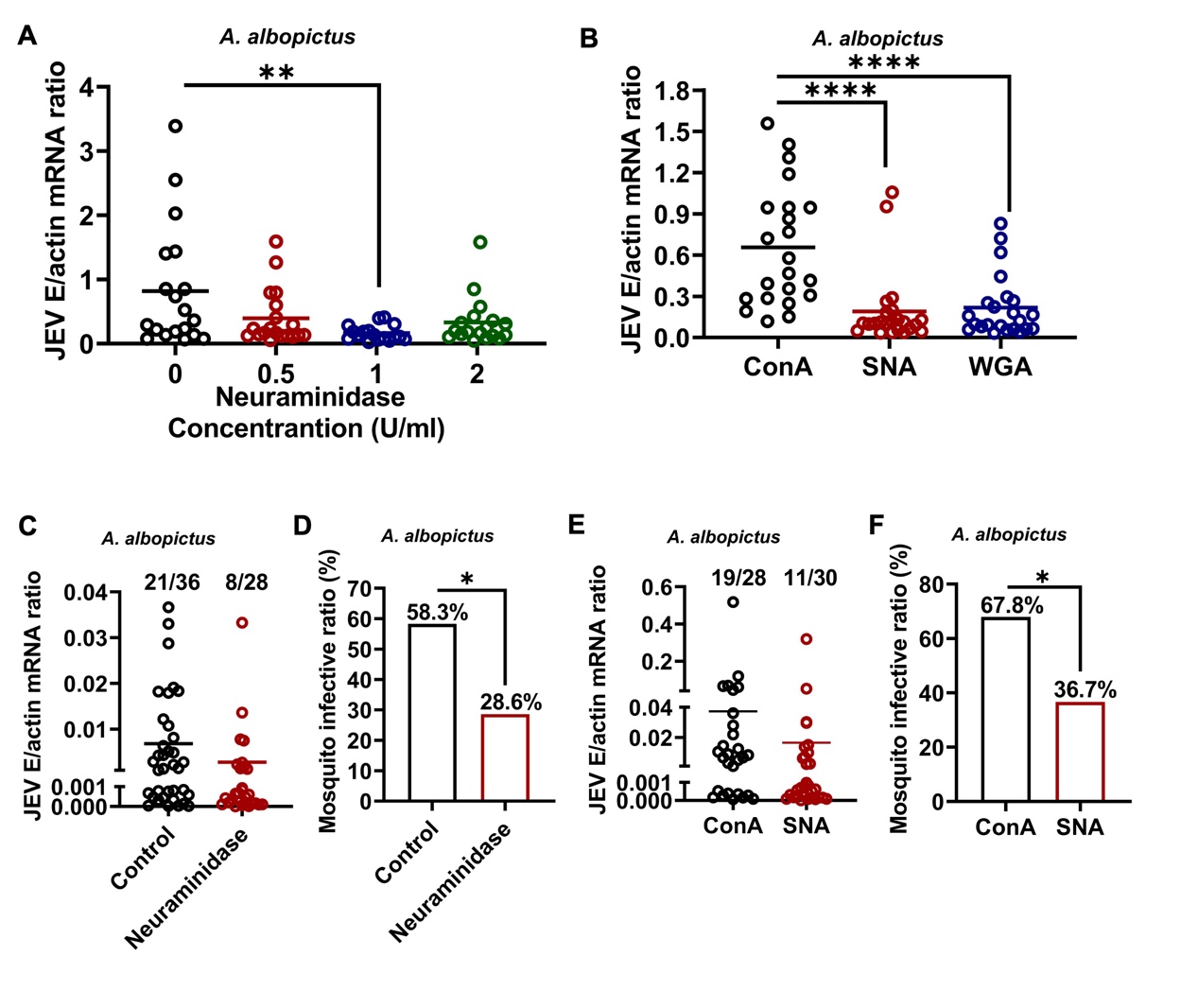
**

**Supplementary Figure 1 Sialic acids play a significant role during JEV infection within *A. albopictus.***

(A and B) Increasing concentrations of neuraminidase or 2 μM/ml lectin were microinjected into *A. albopictus* one day before 10 MID50 JEV infection. The JEV burden was determined by RT-qPCR at 3 days post-infection. (C-F) 1U/ml neuraminidase or 2 μM/ml lectins (ConA or SNA) mixed with fresh mice blood and supernatant from JEV-infected C6/36 cells was used to feed *A. albopictus* via an in vitro blood feeding system. The JEV infection of mosquitoes was determined by RT-qPCR 8 days after a blood meal. (C and E) The number of infected mosquitoes relative to the total number of mosquitoes is shown at the top of each column. Each dot represents a mosquito. The horizontal line represents the mean value of the group. The limit of detection for the viral genome/actin mRNA ratio was 0.001. Gene expression was normalized to the *A. albopictus* actin gene. The data are presented as the mean ± SEM. (D and F) The data at the top of each column represent the percentage of infected mosquitoes. Differences in the infectivity ratio were compared using Fisher’s exact test. (A and B) A nonparametric Mann-Whitney test was used for statistical analyses. **P* < 0.05, ***P* < 0.01, *****P* < 0.0001. The data were combined from two independent experiments.


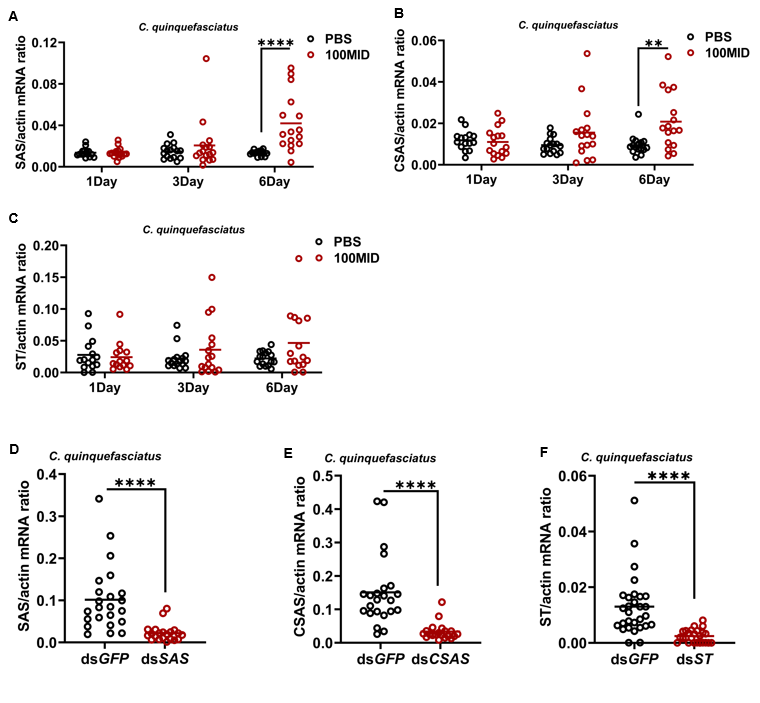


**Supplementary Figure 2 The expression of *SAS*, *CSAS* and *ST* were simultaneously induced by inoculation with JEV.**

(A, B, C) JEV (100 MID50) or PBS was microinjected into *C.* *quinquefasciatus*. The mosquitoes were collected at 1 day, 3 days, and 6 days post-infection. Total RNA was extracted, the *SAS*，*CSAS* and *ST* genes mRNA levels were detected by RT-qPCR. Gene expression was normalized to the *C.* *quinquefasciatus* actin gene. (D, E, F) The *SAS*, *ST* and *CSAS* silencing efficiency in *C.* *quinquefasciatus* was detected by RT-qPCR. Data are represented as mean ± SEM. in each group and analyzed using the nonparametric Mann Whitney test. ***P* < 0.01, *****P* < 0.0001.

**
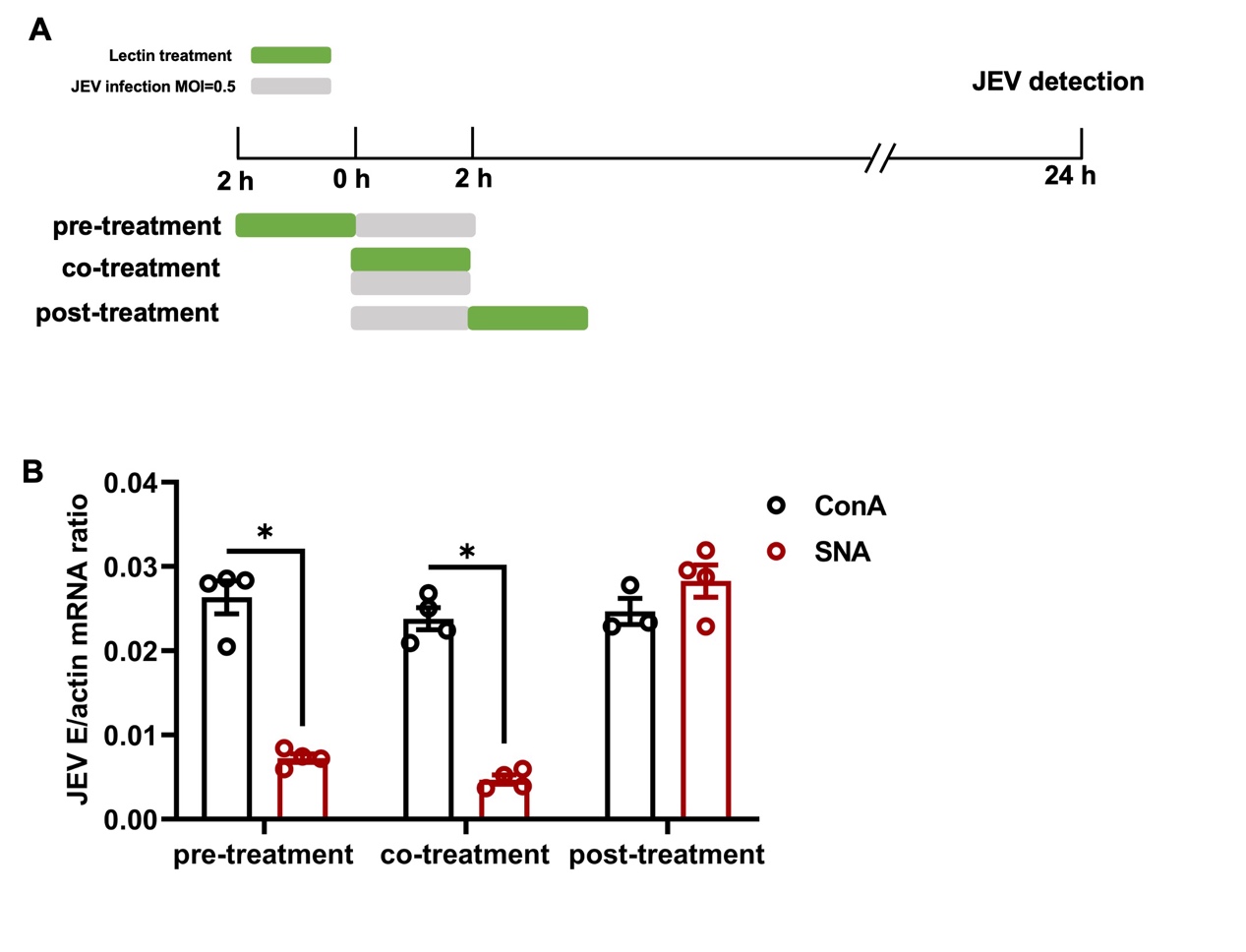
**

**Supplementary Figure 3 Sialic acids on mosquito cell surface play a role of early JEV infection.**

(A) Schematic representation of the study design. C6/36 cells were treated with 1μM/ml SNA in different stages of JEV infection at a MOI of 0.5. 1 μM/ml ConA as a negative control. (B) The infected cells were collected at 24 h post-infection for detection of viral genomes by RT-qPCR. Gene expression was normalized to the *A. albopictus* actin gene. Data are represented as mean ± SEM. in each group and analyzed using the nonparametric Mann-Whitney test. **P* < 0.05. The experiment was biologically repeated three times with similar results.

**
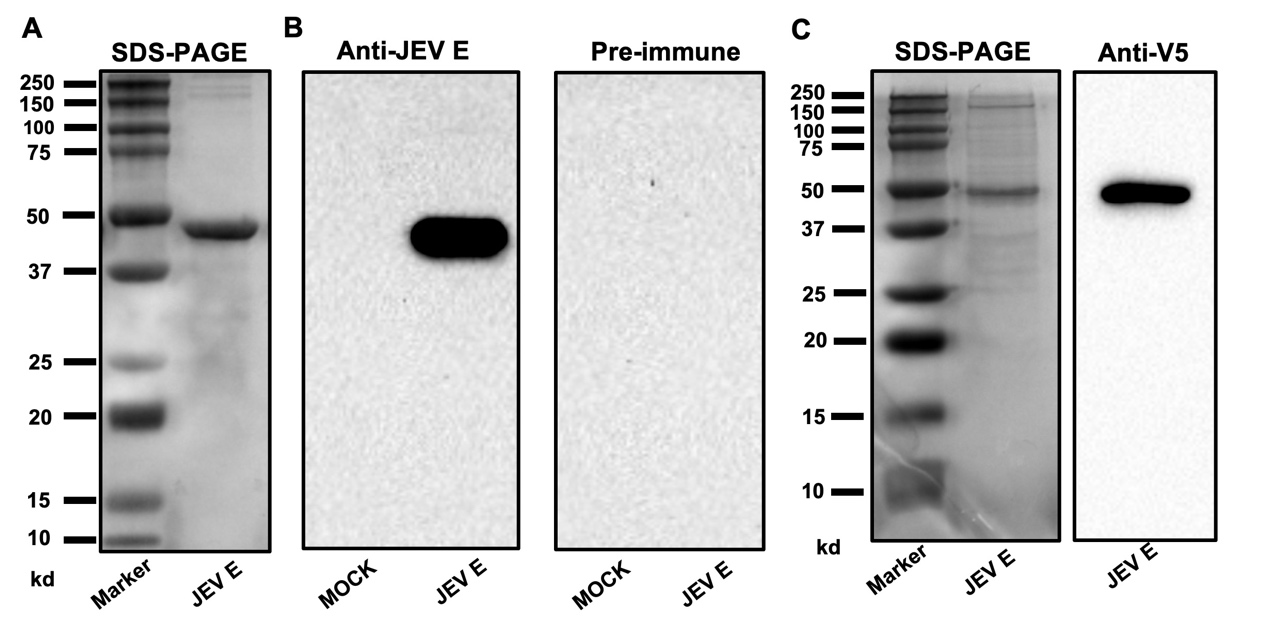
**

**Supplementary Figure 4 Production of murine polyclonal antibodies against JEV E and purification of the JEV E protein from *Drosophila* S2 cells.**

(A) The *JEV E* gene was cloned into a pET-28a (+) expression vector and expressed in *E. coli* BL21 DE3. Recombinant JEV E in inclusion bodies was dissolved in 8 M urea and purified for antibody generation. The purified proteins were electrophoresed on an SDS-PAGE gel, followed by coomassie staining. (B) The antibodies were validated by immunostaining with S2-expressed JEV E protein. The same samples probed by mouse pre-immune antibody served as a negative control. (C) Expression and purification of JEV E protein in *Drosophila* S2 cells. JEV E was cloned into the pMT/BiP/V5-His A expression vector. Recombinant JEV E protein was expressed in *Drosophila* S2 cells and purified using a cobalt-His column, and the purified proteins were electrophoresed on an SDS-PAGE gel, followed by coomassie staining (left). Protein was detected by western blotting with an anti-V5 antibody (right).


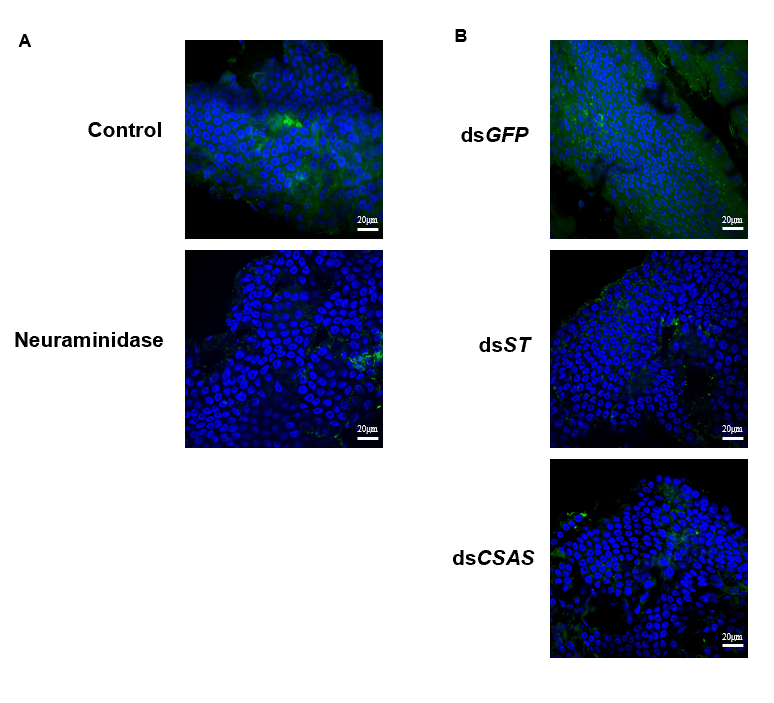


**Supplementary Figure 5 Sialic acids levels in mosquitoes reduced after neuraminidase or dsRNA treatment.**

(A) 1 U/ml neuraminidase were microinjected into *C.* *quinquefasciatus* thoraxes, after one day, the midguts of mosquitoes were dissected to detect the level of sialic acids by immunofluorescence assay. (B) The *CSAS* or *ST* genes was silenced by dsRNA thoracic microinjection in *C.* *quinquefasciatus*. The mosquitoes inoculated with *GFP* dsRNA as mocks. After 3 days, dsRNA-treated mosquitoes were collected to dissect midguts to measure the level of sialic acids by by immunofluorescence assay. Sialic acids were stained with Biotinylated SNA followed by FITC-conjugated streptavidin. Cell nuclei were stained with DAPI. Scale bars, 20 µm.

**Supplementary Table 1 Primers and probe used for qPCR, dsRNA synthesis and genes cloning.**


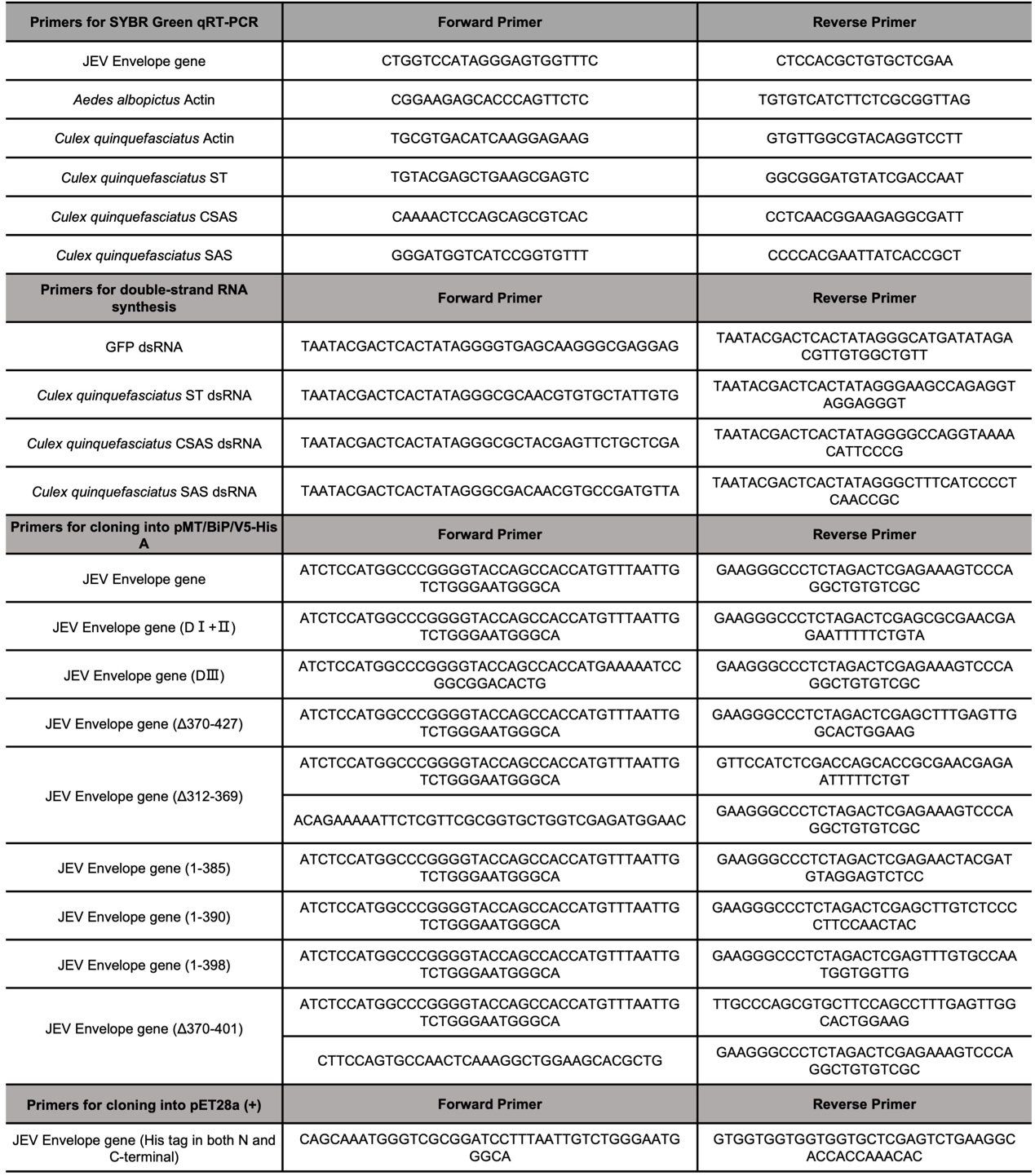

Supplement: Supplemental material — Figures S1 to S5; Table S1. [file jvi.01959-23-s0001.docx]
